# Reduction of Liver Span and Parameters of Inflammation in Nonalcoholic Fatty Liver Disease Patients Treated with Lycosome Formulation of Phosphatidylcholine: A Preliminary Report

**DOI:** 10.1155/2018/4549614

**Published:** 2018-04-01

**Authors:** Ivan M. Petyaev, Pavel Y. Dovgalevsky, Natalia E. Chalyk, Victor A. Klochkov, Nigel H. Kyle, Yuriy K. Bashmakov

**Affiliations:** ^1^Lycotec Ltd, Granta Park Campus, Cambridge CB21 6GP, UK; ^2^Institute of Cardiology, Saratov State Medical University, 12 Chernyshevskogo Str, Saratov 410028, Russia

## Abstract

Twenty-nine newly diagnosed individuals with Nonalcoholic Fatty Liver Disease (NAFLD) remaining on habitual dietary regimen were supplemented with regular or lycosome formulations of phosphatidylcholine (PC) during a pilot, randomized, double-blinded clinical study. After two months of oral PC intake (450 mg daily) the liver size as well as serum levels of hepatic enzymes and markers of inflammation were evaluated by ultrasonography and biochemical analysis. It was shown that there was a statistically significant reduction of medians for the Mid-Clavicular liver size from 16.0 cm (95/5% CI: 17.1/15.5) to 15.1 cm (95/5% CI: 17.2/14.4, *P* = 0.021) in participants ingesting the lycosome-formulated PC (L-PC) whereas regular formulation of PC (R-PC) had only a marginal effect on this parameter (*P* = 0.044). A similar tendency was observed in the Mid-Sternal liver size. Moreover, there was a reduction of medians for ALT values at the end point of the study (*P* = 0.026) after ingestion of L-PC, while R-PC had no statistically significant effect. On the other hand, ingestion of both formulations was accompanied by reductions in values for Inflammatory Oxidative Damage (IOD) and oxidized LDL in serum. However, L-PC had superior activity in these terms, presumably due to the presence of lycopene, a powerful antioxidant, in the L-PC-Lycosome structure. C-reactive protein level was moderately decreased (reduction of medians from 6.5 [95/5% CI: 7.7/5.8] mg/L to 5.1 [95/5% CI: 5.6/4.3] mg/L) only after ingestion of L-PC. The greater efficacy of L-PC seen in NAFLD volunteers may reflect improved bioavailability of PC owing to better protection of the microencapsulated PC from gastrointestinal enzymes and possibly enhanced hepatic delivery of L-PC particles.

## 1. Introduction

Nonalcoholic fatty liver disease (NAFLD) is the most common chronic liver disease in the world, affecting approximately 100 million Americans as well 30% of western and 17% of eastern populations [1–4]. Its major manifestation is hepatic lipid accumulation in the absence of excessive alcohol intake. NAFLD is a multifaceted and uniquely staged disease which includes a spectrum of pathological disorders ranging from simple steatosis without inflammation to hepatic fibrosis and nonalcoholic steatohepatitis—NASH—with further risk of liver carcinoma development [5, 6]. NAFLD is often defined as the main hepatic feature of metabolic syndrome and obesity with a clear causative link to limited physical activity and excessive dietary caloric input achieved by ingestion either of fat, carbohydrates, or both [4, 7]. It has to be mentioned that presence of lipids in liver is a normal phenomenon since the liver is known to be the most active organ of lipogenesis supported by mevalonate and fatty acid synthase pathways tightly controlled by insulin/glucagon ratio and some other hormones [8]. However, fat droplet overaccumulation in hepatocytes due to hormonal imbalance and impaired insulin/glucagon sensitivity is deeply harmful to hepatocyte function and leads to progressive accumulation of free fatty acids, triglycerides, and cholesterol in hepatic and nonhepatic cells [8, 9]. Diminished lipid secretion and enhanced lipid intake as well as reduced betaoxidation of lipids are other features of NAFLD resulting in accumulation of lipids in liver [2, 5].

Since behavioral factors such as excessive caloric intake and limited physical activity are leading etiological factors in NAFLD, life-style interventions are first-choice options in the management of NAFLD [10]. However, as the disease progresses and the vicious cycle of metabolic abnormalities in the liver deepens, life-style interventions become insufficient for reversal of NAFLD. Thus, various pharmaceutical interventions become a necessity to alleviate progression of NAFLD and its hepatic (cirrhosis, hepatonecrosis, and hepatocellular carcinoma) and nonhepatic (cardiovascular disease, type 2 diabetes) complications. As it is today, there are no officially approved effective drugs for NAFLD treatment and pharmacological options are considered for each patient on an individual basis. Among the most widely used drugs for treatment of NAFLD are biguanides (metformin), thiazolidinediones, inhibitors of HMG-CoA reductase (statins), antihypertensive, and antidiabetic drugs [11, 12]. Many authors emphasize that micronutrients may have as profound an effect on NAFLD as pharmaceuticals, suggesting that fiber- and PUFA-enriched hypocaloric diets may have great potential in the treatment of NAFLD [13, 14].

In the present paper we report that a lycosome formulation of phosphatidylcholine (PC), a novel nutraceutical formulation with enhanced hepatic bioavailability of PC, reduces liver span and reverses some metabolic abnormalities in NAFLD patients.

## 2. Materials and Methods

### 2.1. Study Design

The study was conducted by Lycotec Ltd (Cambridge, UK) at the Institute of Cardiology (Saratov's State University, Russian Federation). The study protocol was approved by the local Ethical Committee and registered (ACTRN12615000386538). The study was designed as a pilot, double-blind, interventional, randomized clinical trial involving patients with NAFLD. NAFLD was defined as a state when patients have liver enlargement detectable at the Mid-Clavicular line (>15 cm) confirmed by ultrasonography as well as increased activity of alanine transaminase (ALT, >50 IU/ml). The main objective was to compare the clinical efficacy of two formulations of phosphatidylcholine (conventional versus lycosome-formulated) in NAFLD patients. The primary endpoint of the trial was the size of the liver and activity of liver-specific enzymes (ALT, AST). Secondary end points included parameters of Inflammatory Oxidative Damage (IOD), changes in level of C-reactive protein (CRP), and oxidized low density lipoproteins (LDL-Px).

### 2.2. Patients

Newly diagnosed patients with Nonalcoholic Fatty Liver Disease (NAFLD), males and females, aged between 36–70 years were enrolled in the study.

40 patients were recruited in two clinical groups. 11 patients were not able to complete the study due to various personal reasons not due to the study protocol. 29 patients were able to complete the study. All study participants as well as medical personnel were informed about the purpose of the study and signed a written consent form. All patients underwent scrupulous anamnestic, physical, and laboratory investigation during the enrolment period. Ultrasonography assessment of liver size and biochemical blood parameters were investigated before commencement of the study, at the intermediate time point (1st month after beginning treatment) and at the end point of the study (end of the 2nd month). Physical evaluation included reading the vital signs, anthropometry, determination of body mass index, pulse and arterial blood pressure measurement, and ultrasonography investigation. Among the laboratory tests were determination of fasting serum glucose and lipids (total cholesterol, LDL cholesterol, HDL cholesterol, and total triglyceride levels), determination of ALT and AST levels, total bilirubin, CRP, LDL-Px, and IOD. All volunteers were also subjected to a standard hematological investigation. The volunteers were advised to follow the pattern of habitual food intake to which they were accustomed during the prestudy period. Randomization was performed using a computerized simple randomization protocol. Group assignment of each individual participant was known only to the principal investigator and the independent statistician.

### 2.3. Inclusion/Exclusion Criteria

#### 2.3.1. Inclusion Criteria

1. Liver enlargement detectable at Mid-Clavicular line (>15 cm) confirmed by ultrasonography.
2. Increased activity of alanine transaminase (ALT, >50 IU/ml).
3. Hyperechogenicity, hepatorenal contrast, and liver brightness of the liver tissue as defined by abdominal ultrasonography evaluation.

#### 2.3.2. Exclusion Criteria

Exclusion criteria were as follows: current or anamnestic alcohol use, type 1 or type 2 diabetes mellitus, chronic kidney disease, platelet count < 100.000/mm^3^, pregnancy, hepatitis A, B, or C, cancer, intake of medications affecting liver function or size (metformin, tetracycline, amiodarone, tamoxifen, glucocorticoids, and anabolic steroids), participation in another clinical trial, allergy to tomatoes, unwillingness to participate in the study.

### 2.4. Study Products

Participants were assigned to take either 450 mg of regular PC formulation (Pharmaceutical Grade, Lipoid GmbH, Germany) or 450 mg of PC as Lycosome formulation containing 7 mg of lycopene (Lycotec, Cambridge, UK). Both formulations were taken orally, once a day, with main meal for a period of 2 months.

Lycopene-based lycosomes [15, 16] provide a degree of protection to cargo molecules from stomach acidity and intestinal enzymes increasing thereby the bioavailability of PC and its intestinal absorption rate. Both formulations were dispensed to the participants at the “0” time point and at the intermediate point of the study. Compliance of the participants and their adherence to the protocol was verified at the intermediate and final time points as well as by weekly phone surveys. All individual packages containing PC formulations were labelled with a numerical code and shipped to the study site. None of the volunteers and trial personnel was informed about the study group assignment or the dispensed product specification for the duration of the study period.

All products were to be taken once daily with the main evening meal. The period of administration was 2 months.

### 2.5. BMI, Pulse Rate, and Blood Pressure

Body mass index (BMI) was calculated as described elsewhere. Pulse rate and systolic and diastolic blood pressure were measured three times in the left arm of seated volunteers after 15 minutes of rest. The time between measurements was no less than 2 minutes. The mean value for each parameter was calculated. All parameters were measured in the morning between 8 and 10 am.

### 2.6. Ultrasonography Protocol

Ultrasonography using Philips HD7 XE was conducted in the morning hours in fasting participants by an experienced technician who was unaware of patient allocation in the study groups and previous results of liver size measurements in each patient. The following parameters were taken into consideration and recorded: liver size at Mid-Clavicular and Mid-Sternal lines, parenchymal brightness, liver-to-kidney contrast, deep beam attenuation, brightness of vessel walls, and state of gallbladder wall. All records were saved in numerical files without patient names. Matching and decoding records were performed by the independent statistician and principal investigator.

### 2.7. Blood Collection

Blood was collected from the arm veins of the participants in the morning after fasting. Serum was separated from the clotted mass by centrifugation and aliquots were stored at −80°C prior to analysis.

### 2.8. Laboratory Parameters

Total cholesterol (TC), triglycerides (TG), HDL/LDL cholesterol, glucose, and C-reactive protein (CRP) were measured using a Biosystem A25 automated analyzer (Applied Biosystems, Grand Island, NY) using BioSys kits and calibrators.

### 2.9. Inflammatory Oxidative Damage (IOD)

Serum samples were incubated overnight in 0.05 M PBS acetate buffer (pH 5.6) which would imitate the type of oxidative damage occurring during the release of lysosomes following neutrophil degranulation. The following morning, the reaction was terminated using trichloroacetic acid. The concentration of the end products such as malonic dialdehyde (MDA) and other possible thiobarbituric acid reactive substances (TBARS) was then measured by colorimetric methods [17, 18] using reagents and kits from Cayman Chemical (MC, USA).

### 2.10. Oxidized LDL (LDL-Px)

Activity of serum LDL peroxidase proteins, which include IgG with superoxide dismutase activity, was measured as described previously [19, 20].

### 2.11. Statistics

The results were analyzed as median with 5% and 95% percentiles. Enrolment data are shown as averages with standard deviation. For the assessment of normally distributed parameters, the Shapiro–Wilk method was used. Student's *t*-test was then applied for both paired and unpaired samples. Between-group differences at one time point were evaluated by the Wilcoxon–Mann–Whitney test (continuous variables) and Fisher's exact test (categorical variables). Data analysis was performed using Stata (College Station, TX) SE, version 12.1. All statistical tests were two sided and statistical significance level alpha was set at 0.05 for the analysis.

## 3. Results

As can be seen from Table 1, there was a successful randomization of the participants between the two major groups of the study. All participants enrolled had a moderately increased liver size, as measured by ultrasonography and mild elevation in the serum ALT activity, whereas AST level remained in normal range. Moreover, the participants tended to be overweight with moderately increased BMI and tended to have a borderline increase in both total serum cholesterol and LDL. However, most of them had a normal triglyceride level in serum and were normotensive as well as normoglycemic suggesting altogether an absence of metabolic syndrome. Most importantly, the parameters of liver span in the enrolled participants were moderately increased in both groups of the study as compared to widely reported [21, 22] average values for Mid-Sternal and Mid-Clavicular liver size. Therefore, all enrolled participants had a moderate hepatomegaly.

**Figure 1:**
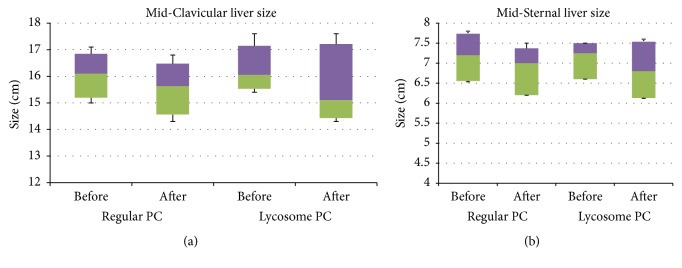
*Box-and-whisker analysis of liver span*. The patients were screened, enrolled, randomized, and treated with regular (PC) or lycosome (PC-Lycosome) formulations of phosphatidylcholine (PC) for 2 months as described in Materials and Methods. Median values with confidence intervals for Mid-Clavicular (a) and Mid-Sternal (b) liver size at time “0” and the end point of the study are shown above.

Figure 1(a) shows the changes of liver span in participants treated with regular and lycosome formulation of PC at the end point of the interventional period. As can be seen, treatment with lycosome-formulated PC caused a statistically significant reduction of the Mid-Clavicular liver size from 16.0 cm (95/5% CI: 17.1/15.5) to 15.1 cm (95/5% CI 17.2/14.4, *P* = 0.021), whereas the regular formulation of PC had a less significant effect on the median values of this parameter at the end point of the study (*P* = 0.044). Reduction of the Mid-Clavicular liver size took place in 12 out of 14 participants treated with lycosome formulation of PC for 2 months, whereas a similar decrease took place in only 9 out of 15 participants treated with the regular formulation of PC (reduction of medians from 16.1 to 15.6 cm). A similar tendency was observed with values reflecting the Mid-Sternal liver size at the end point of the study.

As shown in Figure 1(b), a two-month treatment with lycosome formulation of PC resulted in a small but statistically significant reduction in the Mid-Sternal liver size from 7.2 cm (95/5% CI: 7.5/6.9) to 6.8 cm (95/5% CI: 7.2/6.5, *P* = 0.018) which took place in 12 out of 14 participants. Once again, there was a less significant reduction in the Mid-Sternal liver size parameters (reduction of medians from 7.2 cm to 7.0 cm, *P* = 0.046) of the participants treated with regular PC at the end of interventional period. Changes in liver span observed at the intermediate point of the interventional period (1 month after beginning the study) in both groups were less pronounced (Table 2). Notably, according to the median distribution, even at the intermediate point of the study, the lycosome formulation of PC had a clearly higher impact on reduction of liver size.

**Figure 2:**
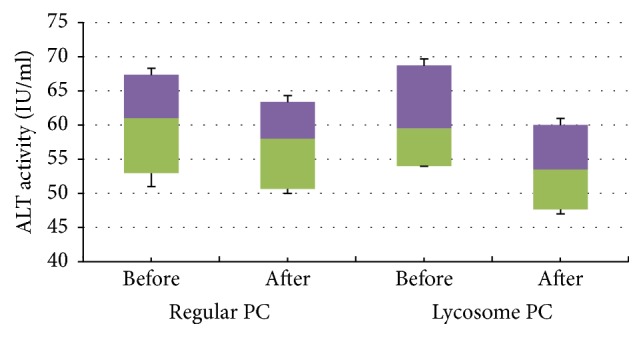
*Box-and-whisker analysis of serum ALT activity*. The patients were screened, enrolled, randomized, and treated with regular (PC) or lycosome (PC-Lycosome) formulations of phosphatidylcholine (PC) for 2 months as described in Materials and Methods. Median values with confidence intervals for serum ALT activity at time “0” and the end point of the study are shown above.

As can be seen in Table 3, all patients enrolled in the study had a normal level of serum AST activity. Its value remained unchanged during the observational period and was not affected by either treatment. However, serum values for ALT were subject to interesting changes. First of all, all participants enrolled in the study had a moderately elevated serum ALT activity. Treatment with regular formulation of PC tends to slightly decrease the median values by approximately 3 units during the course of the study. However, these changes fall short of the range of statistical significance (*P* > 0.05). On the other hand, as can be seen from the box-and-whisker plot analysis (Figure 2), lycosome formulation of PC caused a statistically significant reduction of median ALT values at the end point of the study (*P* = 0.026, after 2 months of treatment). A similar tendency was observed in median distributions (Table 3) at the intermediate point of the study but was not statistically significant (*P* = 0.068, after 1 month of treatment).

Next, Table 4 shows the parameters of inflammation in the serum of the enrolled subjects. As can be seen, Inflammatory Oxidative Damage values decreased in a step-wise manner with both formulations of PC during the course of the study. However, the most prominent decrease took place when the patients were supplemented with lycosome formulation of PC (decline by 55.0% and 67.0% at intermediate and final time points of the study, resp.), whereas the IOD values for regular formulation of PC were reduced less significantly (reductions of medians by 38.0% and 47.2% at the same time points). A similar pattern was seen for oxidized LDL concentration (Table 4). Step-wise decline in LDL-Px medians was seen with both formulations of PC. However, the presence of lycopene in the lycosome formulation of PC caused a much greater reduction of oxidized LDL (by 11.6% and 33.4% after 1 and 2 months, resp.) than was seen with the regular PC formulation (corresponding declines of 5.4% and 12.4%). In contrast, CRP concentration values changed in a more conservative manner. No significant changes in CRP level were seen in both groups at the intermediate time point. However, at the end point of the study there was a clear tendency towards CRP reduction in the case of lycosome-formulated PC (*P* = 0.018). Regular formulation of PC did not affect CRP parameters.

Finally, no significant changes in BMI, serum lipids, fasting glucose levels, or blood pressure parameters were observed.

## 4. Discussion

Phospholipids are a group of lipids essential for the structure and function of biological membranes. Their unique membrane functions are predetermined by the polarity of the phospholipid molecule which has hydrophobic tail (fatty acid residues) and hydrophilic head (phosphate group) connected by a molecule of glycerol [22, 23]. In the case of phosphatidylcholine (PC), an abundant phospholipid in biological membranes, the phosphate group is modified by the addition of choline which confers greater amphiphilicity to the PC molecule. Most of the PC is known to be synthesized in the liver by reciprocal regulation of the CDP-choline (CTP: phosphocholine cytidylyltransferase) and the PEMT (phosphatidylethanolamine N-methyltransferase) pathways [24, 25]. However, due to the enterohepatic circulation, up to 50% of hepatic PC originates from the systemic circulation [24]. The resulting PC is secreted again from the liver into the intestinal lumen with the bile to promote intestinal lipid absorption [26] and/or can be used within the hepatocytes for maintenance of hepatic membranes and synthesis of very-low-density lipoproteins (VLDL) and high-density lipoproteins (HDL).

In the current report we show that continuous oral supplementation with PC formulations ameliorates liver span and reverses some metabolic abnormalities in NAFLD patients. In particular, a two-month oral ingestion of PC leads to a statistically significant reduction of Mid-Clavicular and Mid-Sternal liver size and ameliorates serum ALT level which is an important laboratory manifestation of NAFLD. Notably, the lycosome formulation of PC has greater efficacy in this regard when compared to regular PC.

It should be taken into consideration that there is a solid evidence-based rationale for use of PC supplementation in NAFLD. PC is an important substrate for triglyceride synthesis in the liver. Approximately two-thirds of hepatic TG originates from hepatic phosphatidylcholine [26, 27]. Thus, upregulated triglyceride synthesis and lipid droplet formation in NAFLD livers inevitably lead to the depletion of hepatic PC reserves. Moreover, the majority of steatotic patients display a state of choline deficiency, which limits PC biosynthesis in the liver and is considered to be a major predisposing factor in NAFLD [28]. It should be mentioned that a choline-deficient diet has been used for decades to model NAFLD in animal experiments [29].

Although there is little doubt that PC supplementation might be highly beneficial for NAFLD outcomes, deeper insight is required to explain why the lycosome-PC formulation shows greater efficacy than regular PC in NAFLD patients. Lycosomes are microdelivery particles containing lycopene, a highly active antioxidant carotenoid employed in lycosome structure as a core-forming agent, and PC used as a chaperone [15, 16]. Lycosomes can also be designed to contain various cargo molecules, in particular different amphiphilic pharmaceuticals and nutraceuticals including hydrophobic peptides, HMG-CoA reductase inhibitors, flavanols, and others [30–33]. Lycosome particles are resistant to the acidic environment of the stomach as well as intestinal enzymes and can be partially absorbed in unmodified form. Upon absorption they become incorporated into chylomicrons or VLDL and enter the systemic circulation with further clearance in tissues expressing scavenger receptors and LDL-receptors (liver, adrenals). Moreover, lycopene is a powerful ligand for carotenoid receptors abundantly expressed in the liver and thus hepatic delivery of lycosome particles becomes additionally empowered by the carotenoid uptake system [34]. Therefore, enhanced bioavailability of PC for hepatic metabolism arising from hepatotropism of lycosome particles seems to be a most likely explanation for the greater efficacy of the lycosome formulation of PC in NAFLD patients.

There is another dimension arising from our results. Lycopene is one of the most powerful natural antioxidants with an antiradical capacity exceeding that of beta-carotene and other carotenoids by many times [34]. Additionally, there are multiple reports connecting NAFLD pathogenesis with activation of lipid peroxidation and reactive oxygen species (ROS) formation [35]. Therefore, it could be anticipated that ingestion of lycosome-PC particles, in which lycopene serves as a core-forming compound, may affect some parameters of biological oxidation in NAFLD patients. Indeed, as we have shown above, supplementation with PC-containing lycosomes reduces the levels of Inflammatory Oxidative Damage and oxidized LDL in the serum of NAFLD patients. Interestingly, there was also a small but statistically significant concomitant decrease in serum CRP levels which was seen only in patients supplemented with the lycosome formulation of PC at the end point of the study. Intriguingly, these declines in oxidation and inflammation markers were much less pronounced but still took place in the NAFLD patients supplemented with the regular formulation of PC lacking lycopene. This observation unveils the potential role of PC as an antioxidant molecule whose antiradical activity may develop indirectly via the membrane-stabilizing effect of PC and reveals the potentiation of the antioxidant effects of lycopene and PC within in vivo systems.

And finally, besides its antioxidant properties, lycopene has been shown to inhibit HMG-CoA reductase [36], a rate limiting enzyme of the cholesterol biosynthesis pathway in hepatocytes. Therefore, the greater degree of reduction in liver span in NAFLD patients treated with lycosome formulation of PC could be explained by the direct effect of lycopene on hepatic lipogenesis.

However, our observation has some significant limitations. First of all, there is always an element of subjectivity in sonographic organometry. However, the double-blind approach employed in our work as well as good agreement between clinical and biochemical results confer on us a confidence in the results reported above. Moreover, it should be confirmed by future studies to what extent the liver span reduction seen in NAFLD patients after oral ingestion of the lycosome-formulated PC originates from diminished hepatic fat deposition. Furthermore, the liver span reduction observed in NAFLD patients treated with lycosome formulation of PC may be more pronounced if longer duration of supplementation and dose variations are employed in the future studies. It remains unclear if the beneficial effects of lycosome-formulated PC would be maintained after discontinuation of the treatment. In addition, lack of the placebo group is a significant deficiency of the study. Finally, a larger cohort of patients and addition of representative placebo group are essential to confirm our preliminary results and evaluate other potential benefits of PC supplementation in NAFLD patients.

## Figures and Tables

**Table 1:** Baseline characteristics.

Baseline characteristics of the enrolled patients (mean ± SD)		
Variable	PC-Lycosome	PC
Number of patients	14	15
Males	8	9
Females	6	6
Age	49.1 ± 4.8	51.2 ± 5.7
Light/moderate smokers	4	5
Body mass index	29.5 ± 3.3	28.4 ± 3.8
AST in U/L	39.14 ± 3.77	38.71 ± 3.19
ALT in U/L	60.50 ± 5.54	60.78 ± 5.05
Fasting glucose in mmol/L	5.5 ± 0.49	6.2 ± 0.61
Total cholesterol in mg/Dl	272.0 ± 15.5	262.3 ± 14.2
Triglycerides in mg/Dl	128 ± 12.6	129 ± 12.9
LDL in mg/Dl	220 ± 19.2	207 ± 17.2
HDL in mg/Dl	43.3 ± 3.9	41.4 ± 3.3
Pulse rate per min	71.8 ± 4.2	72.1 ± 5.3
Blood pressure in mm Hg		
Systolic	121.5 ± 7.9	120.8 ± 6.4
Diastolic	75.7 ± 5.2	75.1 ± 6.0
Liver size in cm		
Mid-clavicular line	16.10 ± 0.58	16.2 ± 0.60
Mid-sternal line	7.19 ± 0.43	7.12 ± 0.34

The patients were screened, enrolled, and randomized into two major groups for the study as described in Materials and Methods. Baseline characteristics from the time point “0” of the study were expressed as mean values with standard deviations and are shown above.

**Table 2:** Liver span parameters after treatment with regular and lycosome formulations of PC (medians with 95/5%% CI).

Duration of treatment	Formulations of PC	
	Regular	Lycosome
	Mid-Clavicular size (cm)	
Baseline	16.1 (16.8/15.1)	16.0 (17.1/15.5)
1 month	16.0 (16.8/14.7)	15.7 (17.2/15.0)
2 months	15.6 (16.4/14.5)^*∗*^	15.1 (17.2/14.4)^*∗*^

	Mid-Sternal size (cm)	
Baseline	7.2 (7.7/6.5)	7.2 (7.5/6.9)
1 month	7.1 (7.57/6.2)	7.0 (7.5/6.2)
2 months	7.0 (7.3/6.2)^*∗*^	6.8 (7.2/6.5)^*∗*^

The patients were screened, enrolled, randomized, and treated with regular (PC) or lycosome (PC-Lycosome) formulations of phosphatidylcholine (PC) for 2 months as described in Materials and Methods. Median values with confidence intervals for Mid-Clavicular and Mid-Sternal liver size at time “0” and the end point of the study are shown above;^*∗*^*P* < 0.05 as compared to baseline.

**Table 3:** Serum AST/ALT activity after treatment with regular and lycosome formulations of PC (medians with 95/5%% CI).

Duration of treatment	Formulations of PC	
	Regular	Lycosome
	AST (IU/ml)	
Baseline	38.5 (45.0/34.6)	38.0 (43.0/35.0)
1 month	40.5 (44.3/36.0)	37.5 (42.4/35.0)
2 months	39.0 (43.0/36.0)	36.0 (38.7/35.0)

	ALT (IU/ml)	
Baseline	61.0 (67.3/52.9)	59.5 (68.7/54.0)
1 month	59.0 (65.3/51.6)	57.0 (66.4/51.2)
2 months	58.0 (63.3/50.6)	53.5 (60.0/47.6)^*∗*^

The patients were screened, enrolled, randomized, and treated with regular (PC) or lycosome (PC-Lycosome) formulations of phosphatidylcholine (PC) for 2 months as described in Materials and Methods. Median values with confidence intervals for AST and ALT activity at time “0” and the end point of the study are shown above; ^*∗*^*P* < 0.05 as compared to baseline.

**Table 4:** Inflammatory oxidative damage (IOD) values, oxidized LDL, and C-reactive protein (CRP) levels after treatment with regular and lycosome formulations of PC (medians with 95/5%% CI).

Duration of treatment	Formulations of PC	
	Regular	Lycosome
	IOD (MDA *µ*M/ml)	
Baseline	156.5 (168.7/144.8)	159.0 (169.3/145.6)
1 month	96.5 (110.4/60.4)^*∗*^	71.5 (83.0/57.9)^*∗*^
2 months	74.5 (81.0/67.2)^*∗*^	52.0 (64.8/44.3)^*∗*^

	Oxidized LDL (LDL-Px ELISA × 10^3^)	
Baseline	621.71 (666.25/576.15)	577.21 (605.4/549.00)
1 month	587.5 (634.4/553.0)	510.5 (549.6/483.5)^*∗*^
2 months	547.5 (573.5/478.7)^*∗*^	384.0 (409.0/353.7)^*∗*^

	CRP (mg/L)	
Baseline	6.0 (7.3/5.4)	6.5 (7.7/5.8)
1 month	6.1 (6.6/5.4)	5.6 (6.5/5.0)
2 months	5.8 (6.3/5.3)	5.1 (5.6/4.3)^*∗*^

The patients were screened, enrolled, randomized, and treated with regular (PC) or lycosome (PC-Lycosome) formulations of phosphatidylcholine (PC) for 2 months as described in Materials and Methods. Median values with confidence intervals for IOD, LDL-Px, and CRP at time “0” and the end point of the study are shown above; ^*∗*^*P* < 0.05 as compared to baseline.
